# Erratum to: Fertilization of grapevine based on gene expression

**DOI:** 10.1002/tpg2.20406

**Published:** 2024-02-10

Cheng Zhang, Haifeng Jia, Jingjue Zeng, Tariq Pervaiz, Zhenqiang Xie, Xudong Zhu, Chen Wang

Volume 9, Issue 3, The Plant Genome | First published online July 21, 2016

First published November 1, 2016 | https://doi.org/10.3835/plantgenome2015.09.0083

This article corrects the following:

Tariq Pervaiz's name was misspelled as Tariq Perraiz in the published version.

